# Effectiveness of the Unified Protocol for the transdiagnostic treatment of emotional disorders in online and group format in Argentina: study protocol for a randomized controlled trial

**DOI:** 10.1186/s13063-023-07428-4

**Published:** 2023-10-19

**Authors:** Milagros Celleri, Camila F. Cremades, Martin J. Etchevers, Cristian J. Garay

**Affiliations:** Facultad de Psicología, Universidad de Buenos Aires, Buenos Aires, Argentina

**Keywords:** Unified Protocol, Emotional disorders, Group therapy, Online, Argentina

## Abstract

**Background:**

The Unified Protocol (UP) is a transdiagnostic intervention based on emotional regulation for the treatment of emotional disorders. Its application in individual and group formats has been studied worldwide, obtaining similar results to specific protocols but with a lower drop-out rate and improving the cost–benefit ratio, since a larger number of patients can benefit from it. Moreover, the inclusion of digital technologies in psychotherapy aims to improve the accessibility of treatments, especially since the pandemic of COVID-19 that forced the implementation of treatments through teletherapy increasing its use. To date, no studies have been carried out in Argentina on the application of the UP in a group format and through teletherapy. The aim of the present study is to evaluate the efficacy of the UP in a group format and through teletherapy in the Argentine population.

**Methods:**

A parallel-group, controlled, randomized trial, with pre-post and repeated follow-up measures intergroup design will be conducted. One hundred eighty patients will be randomized to one of the following conditions: an online, group-based UP intervention or a waiting list. The Beck Depression Inventory-II and the Beck Anxiety Inventory will be used to compare primary outcomes and the Beck Hopelessness Scale, Difficulties in Emotion Regulation Scale, Positive Affect and Negative Affect Scale, and Multicultural Quality of Life Index will be administered for secondary outcomes at baseline, post-intervention, and 3 months follow-up. Ad-hoc questionnaires will be used to assess patients’ experiences and treatment satisfaction.

**Discussion:**

The purpose of this trial is to evaluate the efficacy of the online and group application of the UP in the Argentine population, as well as to evaluate the patient’s experience and satisfaction with the treatment. It is expected that the findings of this study will be useful in reducing anxious and depressive symptomatology, will allow us to adapt the UP to our culture, and will improve accessibility to treatment.

**Trial registration:**

ClinicalTrials.gov NCT05275322. Registered on 11 March 2022.

## Administrative information

**Table:** 

Title {1}	Effectiveness of the Unified Protocol for the transdiagnostic treatment of emotional disorders in online and group format in Argentina: study protocol for a randomized controlled trial
Trial registration {2a and 2b}	ClinicalTrials.gov ID: NCT05275322, Registered 11 March 2022, v01
Protocol version {3}	v01
Funding {4}	This research is funded by the University of Buenos Aires, Buenos Aires, Argentina
Author details {5a}	Faculty of Psychology, University of Buenos Aires
Name and contact information for the trial sponsor {5b}	Investigator-initiated clinical trial: C. J. Garay (Principal Investigator) cristiangaray@psi.uba.ar
Role of sponsor {5c}	The funding body will have no role in the design of the study or at any stage of its implementation

## Introduction

### Background and rationale {6a}

Research has established emotional disorders as the mental disorders with the highest prevalence worldwide [1]. In Argentina, the prevalence of anxiety disorders has been estimated at 16.4%, and depression at 8.7%, placing them as the most prevalent disorders among our population [2]. These disorders are highly comorbid to each other and recently have been defined under the umbrella of emotional disorders (ED), as they share common etiological and maintaining mechanisms [3].

These disorders involve significant economic costs for public health given their high prevalence and chronicity. Moreover, access to evidence-based treatments in Argentina is often difficult given the lack of therapists trained in their application [4]. The first-choice treatment recommended by the National Institute for Health and Care Excellence (NICE) for ED is cognitive-behavioral therapy [5]. The Unified Protocol is a transdiagnostic approach focused on common aspects of ED [6] that has shown equivalent efficacy to specific CBT protocols for different disorders across the depressive and anxious spectrum [7].

Given the huge gap in access to treatments, the use of technology in mental health and psychological therapy is expected to increase access to effective treatments in populations that do not have access to these treatments. During the last few years, the application of teletherapy in different disorders has been studied and similar outcomes to face-to-face treatments have been obtained [8–10]. In addition, besides the benefits of the inclusion of digital technologies, group treatments also facilitate greater access to treatments as they offer superior cost-effectiveness [11], which is especially relevant in our population.

The application of the UP in group and face-to-face formats has been studied around the world [12–15], and recently, its online application has begun to be studied in pilot studies [16–18]. In Argentina, its application has been studied in a pilot way in a face-to-face and group format [19] and in a group and online format [20]. To date, this will be the first randomized controlled trial of the Unified Protocol in Argentina.

### Objectives {7}

The aim of the present study is to evaluate the efficacy of the UP in online and group format in the Argentine population. For this purpose, the following research questions have been formulated:

1. Will the Unified Protocol delivered in an online and group format be more effective than the absence of treatment (control group) for anxiety and depressive disorders in the Argentine population?
2. What are the experiences of patients in online and group therapy?

### Trial design {8}

This is the protocol of a parallel-group, controlled, randomized trial, with a pre-post and repeated follow-up measures intergroup design. This trial is framed in the superiority framework.

A two-arm trial is going to be carried out. For this reason, a waiting list has been chosen as a comparator. Participants with low to moderate anxiety/depressive symptoms will be randomly assigned to Group 1: Unified Protocol for Transdiagnostic Treatment of Emotional Disorders (UP) intervention, or Group 2: Wait-List Control group.

The assessment will be conducted at three-time points for Group 1: an initial assessment before being randomized (pretreatment; T0), at the end of the intervention (post-treatment; T1), and 3 months after the completion of the intervention (T2). Group 2 will be assessed on T0 and T1. T2 would not be measured in this group since after T1 they will start treatment.

**Fig. 1:**
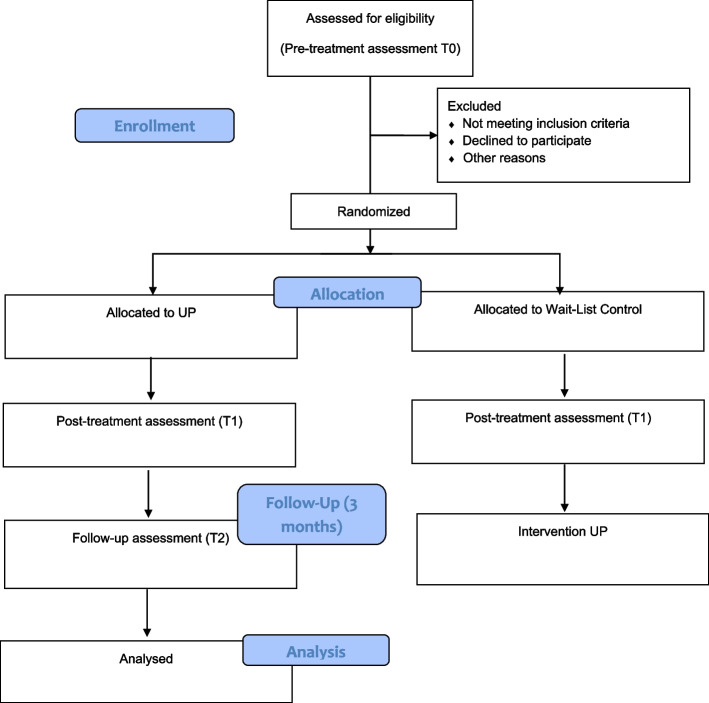
CONSORT flow diagram

CONSORT patient’s flowchart is presented in Fig. 1.

## Methods: participants, interventions, and outcomes

### Study setting {9}

Our research team will conduct this study at the University of Buenos Aires, in Buenos Aires (Argentina), and will be delivered entirely online.

### Eligibility criteria {10}

To participate in this study, participants must fulfill the following inclusion criteria: (1) ≥ 18 years old; (2) submit informed consent; (3) agree to participate in all the scheduled sessions; (4) agree to have the sessions recorded (both audio and video) for monitoring purposes; (5) meet criteria for a primary diagnosis of an anxiety disorder (e.g., panic disorder; agoraphobia; social anxiety disorder; generalized anxiety disorder; unspecified anxiety disorder), or unipolar depression (major depressive disorder; dysthymia) according to DSM-5; (6) not being currently receiving other psychological treatment.

Exclusion criteria will be the following: (1) comorbidity with bipolar disorder, obsessive–compulsive disorder, post-traumatic stress disorder, psychotic disorders, bipolar disorder, eating disorders, borderline personality disorder, substance abuse disorder; (2) presence of suicidal plans and/or acts or having had suicidal plans and/or acts in the last 12 months; (3) having a suicidal ideation score on the Beck Hopelessness Scale (BHS) ≥ 10; (4) being currently receiving psychological treatment or have received psychological treatment within the last year.

All candidates who are not included in the study will be informed of this decision and will be given information on places to seek free treatment.

### Who will take informed consent? {26a}

At the initial screening, participants will receive informed consent from the therapist and will have the opportunity to ask questions about their participation. After that, they will receive the informed consent form and will have to sign it online.

### Additional consent provisions for collection and use of participant data and biological specimens {26b}

In this study, biological samples will not be collected. Participant data will be used only for the research proposes.

### Interventions

#### Explanation for the choice of comparators {6b}

The present study aims to evaluate the efficacy of the application of the Unified Protocol in a group and online format in the Argentinean population. A two-arm trial is going to be carried out. For this reason, a waiting list has been chosen as a comparator.

#### Intervention description {11a}

## Unified Protocol

The UP focuses on different strategies of emotion regulation to address neuroticism, a common factor in all emotional disorders (ED). This intervention intends to address dysfunctional emotional regulation strategies in participants and encourages the use of more adaptive strategies to promote tolerance to intense negative emotions. Participants will receive online, synchronous group therapy based on the UP model for 10 subsequent weeks, with a duration of 90 min for each meeting. After every session, they will receive the chapter of the UP’s Spanish patient’s workbook [6] with the notions addressed in the session and the indications to practice during the week.

Contents of each session:

- Session 1:

Coordinators’ and participants’ introductions. Session framework. Introduction to the approach and ED. Conceptualization of ED and identification of their own intense emotions, aversive reactions, and avoidance efforts.

- Session 2:

Module 1: Motivation for change and commitment to treatment. Describing issues, goals, and steps to accomplish them. Introduction to change motivation. Decision balance exercise.

- Session 3:

Module 2: Understanding emotions. Introduction to the topic of this session (psychoeducation of emotions). Presentation of the three-component model and introduction to the concept of Emotion-Driven Behaviors (EDBs), as well as the antecedents and consequences of emotional experiences.

- Session 4:

Module 3: Emotional awareness training. Introduction to mindfulness. Non-judgment emotional awareness exercises will be carried out during the session, and participants will receive afterwards by email with the taped audio so as to do it during the week. Introduction to emotional induction and practicing mindfulness when facing stimuli that trigger emotions (videos/audio). Introduction to anchoring in the present.

- Session 5:

Module 5: Cognitive flexibility. Introduction to cognitive flexibility. Ambiguous image exercise. Introduction to mind traps and cognitive re-evaluation exercise during the session.

- Session 6:

Module 5: Emotional avoidance and EDBs. Introduction to emotional behaviors. Introduction to EDBs and avoidance types. A cognitive suppression exercise will be carried out during the session. Working with alternative actions.

- Session 7:

Module 6: Physical sensations tolerance. Introduction to interoceptive exposure. Interoceptive exposure exercises during the session: hyperventilating, running on the spot, jumping, breathing through a straw, and twirling.

- Sessions 8 and 9:

Module 7: Emotional exposure. Introduction to the exposure to intense emotions. Building the exposure hierarchy. Introduction to the emotional exposure form.

- Session 10:

Module 8: Maintenance of treatment gains and relapse prevention. Introduction to relapse prevention and plans for future practice. Closure and group farewell among therapists and participants.

### Wait-List Control

Participants in the waiting list control will receive the same intervention as described above after the end of the group intervention. Participants on the waiting list will complete the same assessments as the intervention group (T0, T1), except the last one (T2).

### Criteria for discontinuing or modifying allocated interventions {11b}

In this study, the criteria for discontinuing the intervention will be (1) participants who fail to attend two or more sessions, (2) participants who have current suicidal plans or acts, and (3) participants who request to withdraw from the trial.

Participants who have current suicidal plans or acts will be withdrawn from the trial and will receive individual care.

### Strategies to improve adherence to interventions {11c}

The therapists will encourage the importance of attending scheduled sessions and completing the exercises learned between sessions. After each session, an email will be sent to remind this. If a participant is absent from one session, a member of the research group will contact him/her by phone.

### Relevant concomitant care permitted or prohibited during the trial {11d}

In this trial, participants who receive another psychological intervention during their participation in the study will be excluded. Psychopharmacological interventions are permitted with the regard that the dose dosage remains stable during the trial.

### Provisions for post-trial care {30}

No potential harm is expected from this study.

### Outcomes {12}

#### Primary outcomes

In this study, participants are expected to show improvement in anxiety (BAI), and depression (BDI II) at post-treatment (T1) compared to pre-treatment (T0), and this result is expected to remain stable for 3 months (T2). In Wait-List Control, it is expected that there would not be a significant improvement in these variables at T1 compared to T0.

The clinical cut-off point for these scales in the Argentine population is 10 points. A cut-off score ≥ 10 in the BDI and BAI indicates clinical anxiety or depression (mild, moderate, or severe). A score ≤ 10 indicates that symptoms of anxiety or depression are not clinically relevant.

#### Secondary outcomes

Participants are expected to show improvement in emotion regulation (DERS), quality of life (MQLI), positive and negative affect (PANAS), and hopelessness (BHS) at post-treatment (T1) compared to pre-treatment (T0), and this result is expected to remain stable for 3 months (T2). No significant changes in Wait-List Control on these variables are expected at T1 compared to T0.

#### Participant timeline {13}

**Fig. 2:**
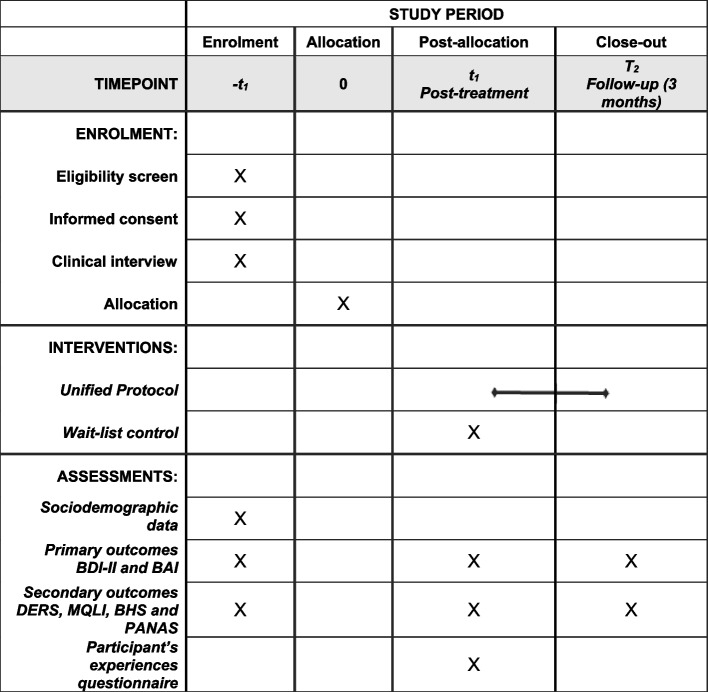
Schedule of enrolment, interventions, and assessments

The schedule of enrolment, interventions, and assessments are shown in Fig. 2.

#### Sample size {14}

The sample size has been estimated based on a meta-analysis of previous studies of the application of the Unified Protocol in emotional disorders compared to a control group [21]. These studies showed a medium effect size (0.45 g* hedges*) on post-treatment measures. A median effect size (0.5) was assumed to detect differences between the intervention and the control group. G*Power software was used to calculate the sample size. For two groups, with a statistical power of 0.80, and an alpha of 0.05, it is estimated that a total of 150 participants will be required (75 for each condition). Given that previous studies have reported a dropout rate between 15 and 20% [22], the total sample required will be 180 participants, 90 for each group.

#### Recruitment {15}

Participants in this study will be recruited via flyers on social media (Facebook and Instagram), from references from colleagues (university faculty members, clinicians, and researchers), and via flyers/posters on the University of Buenos Aires.

## Assignment of interventions: allocation

### Sequence generation {16a}

Simple randomization will be used in this study. Participants who met the inclusion criteria will be randomized to either treatment condition (UP) or Wait-List Control, using specific online software (https://www.randomizer.org/). Ratio allocation will be 1:1 (approximately).

### Concealment mechanism {16b}

Treatment group assignment to participants or therapists cannot be concealed due to the nature of psychotherapy interventions. The allocation will be made by a member of the research team who will not be involved in the screening or group treatment.

### Implementation {16c}

A member of the research team who will not be involved in patient recruitment, assessment, or delivering the treatment will perform randomization and allocation.

## Assignment of interventions: blinding

### Who will be blinded {17a}

Due to the nature of this intervention, treatment allocation cannot be blinded to the therapist or participants. Participants will not receive details about the research aims.

### Procedure for unblinding if needed {17b}

This trial is not blinded. Unblinding the allocation will not be necessary at any point.

## Data collection and management

### Plans for assessment and collection of outcomes {18a}

#### Diagnosis (DSM-5 criteria)

A semi-structured online interview will be carried out in order to assess major psychiatric disorders according to the DSM-5 [23]. All the clinicians involved in this work were trained in clinical interviews and psychopathology.

### Demographic and satisfaction/acceptability information

#### Ad-hoc socio-demographic questionnaire

An ad-hoc sociodemographic questionnaire was developed in order to assess age, self-perceived gender, place of residence, and educational level.

#### Ad-hoc acceptability and perceived satisfaction with the treatment questionnaire

An ad-hoc questionnaire was developed with the aim of evaluating the experience with the treatment and the acceptability of the intervention. For this purpose, 9 items were constructed with a Likert-type response format from 1 to 10. This questionnaire was based on the previous study of Osma et al. [14].

### Primary outcome measures

#### ***Beck Depression Inventory-II (BDI II; ***[24]***)***

The BDI II is a questionnaire that was designed to measure the severity of individuals’ depressive symptoms. It contains 21 items that explore the typical symptoms of a major depressive episode. Each item presents 4 alternatives, ranging from 0 (not at all) to 3 (severe, almost intolerable), on a Likert-type scale. It was validated and adapted to the Argentine population, with an appropriate internal consistency (Cronbach’s alpha coefficient of 0.88.)

#### ***Beck Anxiety Inventory (BAI; ***[25]***)***

It is a scale proposed to measure the severity of anxiety symptoms. It is composed of 21 items and each of these items scores from 0 (not at all) to 4 (I could hardly bear it) on a Likert scale. It has been validated in the Argentine population and shows a good internal consistency (Cronbach alpha coefficient of 0.93).

### Secondary outcome measures

#### ***Beck Hopelessness Scale (BHS; ***[26]***)***

This instrument was created to measure pessimism and hopelessness. It consists of 20 items with a true or false response format, with a possible score that ranges from 0 to 20, with higher scores indicating the presence of strong hopelessness. This instrument was previously validated and adapted to Argentina (Cronbach's alpha coefficient of 0.78).

#### ***Difficulties in Emotion Regulation Scale (DERS; ***[27]***)***

This scale measures the degree to which individuals use different emotional regulation strategies. The adapted scale consists of 30 self-administered items with a five-choice Likert-type scale that ranges from 1 (almost never) to 5 (almost always). This scale presents a robust Cronbach's alpha coefficient of 0.94.

#### ***Multicultural Quality of Life Index (MQLI; ***[28]***)***

This scale was created to measure the quality of life in a brief, multicultural, and multidimensional way. In this self-administered 10-item instrument, each item addresses an area of functioning on a Likert-type scale ranging from 1 (poor) to 10 (excellent). All these items are summarized to obtain a Global Quality of Life Index. This instrument was validated and adapted to the Argentine context with a Cronbach’s alpha coefficient of 0.85.

#### ***Positive and Negative Affect Schedule (PANAS; ***[29]***)***

This inventory has been designed to measure affect in a dimensionally way (negative affect and positive affect). It comprises four dimensions: trait positive affect (five items), trait negative affect (five items), state positive affect (five items), and state negative affect (five items). Each item uses a Likert-type scale ranging from 1 (not at all) to 5 (very much or completely). It was validated in Argentina with a good internal consistency (Cronbach’s alpha coefficient of 0.73).

#### Plans to promote participant retention and complete follow-up {18b}

Upon completion of the intervention, participants will be contacted by e-mail and phone calls to encourage them to complete the follow-up.

#### Data management {19}

All data obtained from the screening will be recorded automatically by the secured platform. Patients will accept the informed consent and will be contacted by a researcher of the team for the online interview. The members of the research team in charge of the diagnoses interviews will be trained and will be provided with a semi-structured interview based on DSM-5 in order to unify the criteria. Interview notes will be stored in a secure office at the University of Buenos Aires.

After that, patients’ data will be anonymized by a unique ID code on the dataset and will be stored in a secure server and the computer will have a password to be accessed only by the principal investigators.

#### Confidentiality {27}

Before entering the study, participants will complete the initial screening and the informed consent. Participants’ information will be encoded with a unique ID number in order to maintain confidentiality. The database will be protected on a computer with password security on a secured platform. Only the principal investigator in the research team will have access to the data.

#### Plans for collection, laboratory evaluation, and storage of biological specimens for genetic or molecular analysis in this trial/future use {33}

N/A, no biological samples will be collected.

### Statistical methods

#### Statistical methods for primary and secondary outcomes {20a}

Statistical analysis will be performed with the latest version of SPSS for Windows or R. Sociodemographic variables will be analyzed using descriptive statistics and will be presented in tables. Means and standard deviations will be calculated for numerical variables and frequencies and percentages for categorical variables. The chi-square test and *t-test* will be used in order to examine if group participants differ in their baseline characteristics.

For primary and secondary outcomes, longitudinal changes in each group (T0, T1, T2) and changes between groups will be calculated with a mixed linear model (MANOVA). The effect size for pre and post-treatment and between groups will also be calculated.

A Bonferroni-Holm correction will be conducted as a multivariate correction method to address the issue of multiple comparisons.

#### Interim analyses {21b}

No interim analysis will be carried out in this trial.

#### Methods for additional analyses (e.g., subgroup analyses) {20b}

In this study, additional analyses have not been planned.

#### Methods in analysis to handle protocol non-adherence and any statistical methods to handle missing data {20c}

To address protocol non-adherence, participant education will emphasize the importance of adhering to the protocol, while clear instructions and materials will be provided. Investigators and research staff will receive training and ongoing support. For this, monitoring and supervision procedures will be implemented to detect and correct deviations. Standardization of intervention and data collection procedures across sites will be ensured. Moreover, motivational strategies will be carried out to encourage adherence while barriers to adherence will be identified and addressed.

Missing data will be imputed using the random forest method.

#### Plans to give access to the full protocol, participant-level data, and statistical code {31c}

The corresponding author will provide access to the anonymized data upon reasonable request.

## Oversight and monitoring

### Composition of the coordinating center and trial steering committee {5d}

In this trial, members of the research team and the principal researcher will be in charge of and responsible for monitoring the research.

### Composition of the data monitoring committee, its role and reporting structure {21a}

In this trial, a data monitoring committee (DMC) is not planned due to not expecting any harm to participants.

### Adverse event reporting and harms {22}

In this study, no harm or adverse events are anticipated (a pilot study has been conducted previously). However, in case of any adverse events, psychotherapists will take appropriate actions to address them. Patients will be encouraged to express any discomfort associated with the intervention and will be provided with information on public places for consultation/treatment.

### Frequency and plans for auditing trial conduct {23}

The School of Psychology of the University of Buenos Aires does not have a department dedicated to auditing current studies. However, the research team meets weekly to monitor the study and its procedures.

### Plans for communicating important protocol amendments to relevant parties (e.g., trial participants, ethical committees) {25}

All changes in the protocol must be approved by the University of Buenos Aires. If any changes to the protocol are required during this trial, a new protocol will be designed and submitted to the ethics committee for approval before implementation.

### Dissemination plans {31a}

The results of this study will be disseminated in several ways: (1) in scientific conferences and meetings, (2) in university extension activities, and (3) in peer-reviewed indexed journals in the field of clinical psychology.

## Discussion

The first aim of the present research is to evaluate the efficacy of the group and online application of the UP in the Argentine population. So far, this is the first randomized controlled trial that assesses the efficacy of a cognitive-behavioral transdiagnostic intervention (in this case, the Unified Protocol) compared to a waiting list condition in a group setting and through teletherapy in our country. These studies are relevant in the Argentine context because cognitive-behavioral interventions are usually developed in high-income countries involving other cultural contexts and little is known about the necessary adaptations for such interventions to be effective in Latin American and middle- or low-income countries.

Moreover, being a transdiagnostic and group-based intervention, it allows treating diverse patient populations (anxiety disorders, depression, and related disorders) simultaneously. This saves resources and its implementation in the public health system is particularly relevant in our population given that access to evidence-based therapies is scarce.

On the other hand, it is expected that remote treatments via teletherapy will increase the accessibility of treatments. This is important in our population, as access to evidence-based treatments and therapists trained in these treatments is often difficult in rural contexts or places far from large urban centers.

Therefore, it is expected that the study of this protocol and its subsequent implementation in health care services will have several advantages. In addition to being a group application (more patients receiving treatment in less time), there are the added benefits of virtuality: people who live far away or who do not have access to nearby care facilities can attend these sessions.

Another objective of this trial is to evaluate patients’ experiences of the intervention, which will allow us to decide what adaptations are necessary when applying an intervention of these characteristics in our population. It is hoped that adapting the treatment to patients’ preferences will improve adherence and reduce dropout rates.

Finally, this study has some limitations. On the one hand, the fact that participants were recruited via social media can be considered a bias. Having a sample of subjects who interact with our social networks or know someone who does decreases the generalizability of the results. At the same time, although the objective of the inclusion of digital technologies in mental health is to increase access to treatment, participation in the study requires a device with Internet access, which may mean that vulnerable populations without access to digital technologies or the Internet are also excluded from access to these treatments.

## Trial status

This is the first version of the protocol v01 (published 11.03.2022). The recruitment process started on April 01, 2022, and is expected to be completed in December 2023.

## Data Availability

In this study, only members of the research team will have access to the data.

## Abbreviations

UP: Unified Protocol
ED: Emotional disorders
DSM-5: Diagnostic and Statistical Manual of Mental Disorders—Fifth Edition
BDI-II: Beck Depression Inventory-II
BAI: Beck Anxiety Inventory
BHS: Beck Hopelessness Scale
DERS: Difficulties in Emotion Regulation Scale
MQLI: Multicultural Quality of Life Index
PANAS: Positive Affect and Negative Affect Scale
